# Targeting the PD-1/PD-L1 Axis in Human Vitiligo

**DOI:** 10.3389/fimmu.2020.579022

**Published:** 2020-11-06

**Authors:** Marcella Willemsen, Cornelis J. M. Melief, Marcel W. Bekkenk, Rosalie M. Luiten

**Affiliations:** ^1^ Department of Dermatology and Netherlands Institute for Pigment Disorders, Amsterdam University Medical Centers, location AMC, University of Amsterdam, Cancer Center Amsterdam, Amsterdam Infection & Immunity Institute, Amsterdam, Netherlands; ^2^ ISA Pharmaceuticals, Leiden, Netherlands

**Keywords:** vitiligo, programmed cell death 1 receptor, B7-H1 antigen, immune tolerance, autoimmunity

## Abstract

Autoreactive CD8^+^ T cells play a pivotal role in melanocyte destruction in autoimmune vitiligo. Immunotherapy for melanoma often leads to autoimmune side-effects, among which vitiligo-like depigmentation, indicating that targeting immune checkpoints can break peripheral tolerance against self-antigens in the skin. Therapeutically enhancing immune checkpoint signaling by immune cells or skin cells, making self-reactive T cells anergic, seems a promising therapeutic option for vitiligo. Here, we review the current knowledge on the PD-1/PD-L1 pathway in vitiligo as new therapeutic target for vitiligo therapy.

## Background

Vitiligo is a common, acquired skin disease characterized by loss of epidermal melanocytes, resulting in sharply, depigmented macules (1). While there is still an extensive debate on the initiating steps, experts agree on immune-mediated melanocyte destruction, with melanocyte-specific CD8^+^ T cells being able to kill melanocytes (2–4) and anti-melanocyte antibodies found in the sera of patients with vitiligo (5). Especially IFN-γ holds a crucial role in the pathogenesis of vitiligo (6). Moreover, regulatory T cells are impaired, both in numbers and function, in patients with vitiligo (7–9). Recently, tissue-resident memory T (T_RM_) cells were shown to have a prominent role in disease development and flare-up in human vitiligo. Vitiligo affected skin was shown to be enriched for CD8^+^ T_RM_ cells compared to healthy, unaffected donor skin, together indicating immune disturbance in patients with vitiligo (10, 11).

Current treatment modalities aim to block melanocyte destruction and/or induce repigmentation of the skin. Topical corticosteroids or immunosuppressants have shown good outcome, while this might not be the most effective approach (12). Moreover, clinical efficacy of current treatment modalities is not satisfactory and, therefore, new therapeutic strategies should be evaluated and tested for vitiligo patients.

Immune checkpoints play a pivotal role in immune evasion by tumors and immunotherapy for melanoma shows that targeting of immune checkpoints is sufficient to break peripheral tolerance and re-activate melanoma-specific cytotoxic T cells in a proportion of patients. Clinically opposite to melanoma, vitiligo patients might benefit from induced peripheral tolerance, turning the autoimmune response into an anergic response. In this review, we therefore propose that exploiting the PD-1/PD-L1 axis may be effective as a treatment strategy for vitiligo.

## Autoimmunity and Tumor Immunity

Similarities exist between skin autoimmunity and tumor immunity, observed in vitiligo and melanoma. Immunization of mice bearing B16 melanoma with gp100 antigen and tumor-specific CD8^+^ T cells resulted in tumor destruction, but also caused vitiligo development (13). Similarly, in melanoma patients, vitiligo can occur spontaneously or upon immunotherapy. Immunotherapy for melanoma often leads to autoimmune side-effects, including vitiligo-like depigmentation. This depigmentation is caused by activated anti-melanoma immunity, that targets not only malignant cells, but also healthy melanocytes and correlates with prolonged survival (14–18). Conversely, vitiligo patients have 3-fold less risk of developing melanoma during life (19, 20). Nevertheless, this risk is suggested to be influenced by individual skin type (21). Melanocytes are far more protective in dark skin types and, therefore, loss of melanocytes leads to a greater skin cancer risk in these patients. Contrary, in patients with light skin types, loss of protective melanocytes seems to be less important and increased immunosurveillance contributes to an overall decreased risk of developing skin cancer. Melanoma susceptibility in vitiligo patients may, therefore, be adjusted for race. Also, immunotherapy for melanoma using a skin-depigmenting compound induced local and systemic anti-melanoma immunity (22). Conversely, a proportion of metastatic cutaneous melanoma patients develop vitiligo-like depigmentation (16, 17). Considering this melanoma/vitiligo relationship, vitiligo patients could benefit from anti-melanocyte tolerance, observed in melanoma, and exploiting immune checkpoints might influence peripheral tolerance against self-antigens in the skin.

## PD-1/PD-L1 signaling

### PD-1/PD-L1 Pathway

Programmed cell death 1 (PD-1 or CD279) is an inhibitory cell-surface molecule that suppresses T cells. PD-1 has emerged as a key player in immune regulation and is constitutively expressed by regulatory T cells and can be, upon activation, expressed by effector T cells, natural killer cells, and B cells (23). Immune suppression requires PD-1 binding on T cells to PD-1 ligands (PD-L1 and PD-L2) on other cell types. PD-L1 is constitutively expressed by various immune cells, e.g., lymphocytes, DCs and macrophages, and its expression can be induced on non-immune cells, including cancer cells. Ligation of PD-1/PD-L1 represses the activation and function of autoreactive T cells, inhibits their proliferation and induces apoptosis, thereby regulating both central and peripheral tolerance to hamper and regulate inflammatory responses and autoimmune diseases (24).

### Melanoma Immunotherapy

Signaling *via* the PD-1/PD-L1 axis is commonly involved in tumor immune evasion. In melanoma, tumor-associated PD-L1 can functionally suppress T cell responses to melanoma and promote T cell apoptosis (25). Consequently, PD-L1^+^ melanomas have been identified as a subset that tends to be more aggressive and is associated with poor prognosis (26).

Targeting these immune checkpoint molecules has become one of the therapeutic options (27). PD-1-targeting monoclonal antibodies have been approved for clinical use and are currently among the first-line treatment options for advanced melanoma patients and many other cancers (28, 29). In a fraction of melanoma patients (ranging from 3.4 to 28%), vitiligo-like depigmentation (or melanoma-associated leukoderma) occurs as an adverse effect of immune checkpoint inhibition, indicating the breaking of tolerance to melanocytic self-antigens (30, 31). Nevertheless, skin depigmentation has now been reported in anti-PD-1/PD-L1 treated patients with other metastatic cancers as well (32–37). Skin depigmentation is significantly associated with a favorable prognosis in melanoma patients (17, 18). Likewise, PD-L1-targeting antibodies, e.g., atezolizumab, are currently being investigated in metastatic melanoma patients (38). Considering vitiligo-like depigmentation in (melanoma) patients receiving anti-PD-1 monotherapy, vitiligo patients may benefit from induced peripheral tolerance to self-proteins by exploiting the T cell immunosuppression mediated by these molecules.

### PD-1/PD-L1 in Autoimmune Diseases

Emerging evidence demonstrates that impaired PD-1/PD-L1 function is involved in a variety of autoimmune diseases, among which type 1 diabetes, inflammatory bowel diseases, and rheumatoid arthritis (39). Manipulating PD-1/PD-L1 signaling appears to elicit significant outcomes in disorders with aberrantly-regulated immune system function. More specifically, PD-L1-expressing DCs can induce anergy of otherwise active T cells and, thereby, hinder induction of autoimmune encephalomyelitis, an animal model of multiple sclerosis (40). Also, intravenous injection of recombinant adenovirus expressing full-length mouse PD-L1 (Ad.PD-L1) gene can suppress lupus-like syndrome in BXSB mice, when combined with anti-ICOSL(B7h) antibody to block ICOS-mediated co-stimulation (41). In this study, it was suggested that the protective effect of Ad.PD-L1 was through suppression of autoreactive T cells at the target organ. Furthermore, severe psoriatic inflammation was induced in PD-1-null mice and PD-L1-Ig fusion protein was shown effective in inhibiting inflammatory skin γδ-low T cell activity *in vitro* (42), indicating the rationale to test the therapeutic potential of increasing PD-1/PD-L1 signaling in skin autoimmunity. Raising our knowledge on PD-1/PD-L1 functions and signaling may thus enable us to develop new therapeutic strategies to manipulate the inhibitory function of PD-1.

## PD-1/PD-L1 in Vitiligo

### PD-1/PD-L1 Expression in Vitiligo

While abundantly studied in melanoma, the PD-1/PD-L1 axis in vitiligo has received far less attention thus far, but deserves exploration as a therapeutic target. Similarly to melanoma cells, PD-L1 can be expressed by melanocytes, especially in the case of inflammatory responses (43). PD-L1-expressing melanocytes co-localize with infiltrating immune cells and PD-L1 strongly correlates with immune cell infiltration in both nevi and malignant melanoma. Also, CD45 and IFN-γ mRNA were detected in PD-L1^+^ melanomas, while IFN-γ could not be detected in PD-L1^-^ tumors. Expression of PD-L1 can be upregulated on various cell types upon stimulation with pro-inflammatory cytokines, including IFN-γ (44). Although IFN-γ-producing CD8^+^ T cell are abundantly present in lesional vitiligo skin, cytokine-induced PD-L1 expression might not be occurring or insufficient in driving a negative feedback loop.

Vitiligo patients also have increased PD-1 expression on peripheral CD3^+^ CD4^+^ and regulatory T cells (45) compared to healthy individuals, suggesting involvement of PD-1 in disease regulation and pathogenesis. Blocking PD-L1 signaling *in vitro* led to expansion of regulatory T cells (46), implicating that PD-1/PD-L1 engagement may negatively regulate regulatory T cell function. The authors, therefore, hypothesized that PD-1^+^ regulatory T cells may become exhausted by PD-L1-expressing autoreactive T cells, hence leading to a deficiency of regulatory T cells in vitiligo (45). PD-L1 expression by autoreactive T cells, however, has not been shown. Similarly, vitiligo patients show increased PD-1 expression on CD8^+^ T cells compared to healthy donor CD8^+^ T cells (47), implying that PD-1 expression is due to excessive activation of autoreactive CD8^+^ T cells. PD-1 expression on CD8^+^ T cells appears to positively correlate with disease activity (47), suggesting a more vigorous attempt to control the inflammatory situation by increasing PD-1 expression. *In situ*, PD-1 is significantly expressed in marginal and lesional infiltrates when compared to non-lesional skin in patients with active non-segmental vitiligo (48). Because no CD4/PD-1 and CD8/PD-1 double staining was done, it remains unstudied which skin T cells express PD-1. Together, these results suggest a role for PD-1/PD-L1 in the lack of peripheral tolerance in vitiligo, providing a rationale to target this axis in vitiligo treatment.

Vitiligo is a polygenic disorder, implying simultaneous contributions of multiple genetic risk factors and environmental triggers. Large-scale genome-wide association studies have identified approximately 50 genetic loci that contribute to vitiligo risk, of which a large fraction encode proteins involved in immune regulation (49–52). To date, no single-nucleotide polymorphisms in the *PD-1* (*PDCD1*) or *PD-L1* (*CD274*) gene has been reported in human vitiligo. Nevertheless, *PD-1* polymorphisms that give a higher risk of developing other autoimmune disorders, among which rheumatoid arthritis and systemic lupus erythematosus, have been reported (53). Additionally, microsatellite polymorphisms in the *CTLA*-4 gene, encoding the CTLA-4 immune checkpoint molecule, have been demonstrated in European-derived vitiligo patients (54). Likewise, a missense polymorphism in the *PTPN22* gene has been associated with susceptibility to autoimmune disorders, including vitiligo (55). Lymphoid protein tyrosine phosphatase (LYP), encoded by the *PTPN22* gene, is an important downregulator of T cell activation. Hypothetically, patients carrying a *PTPN22* polymorphism initially might be less sensitive to T cell inhibition (e.g., by PD-1/PD-L1 signaling), since the LYP missense polymorphism drastically reduce the binding of LYP to C-terminal Src kinase (CSK) *in vitro*. As a result downregulation of T cell activation is disrupted so that T cells lacking the LYP-CSK complex remain hyper-reactive.

### Targeting PD-1/PD-L1 in Vitiligo

Therapeutically enhancing immune checkpoint signaling on self-reactive T cells, making them anergic, seems a promising therapeutic option for vitiligo. Maio et al. (2018) studied PD-1/PD-L1 signaling as a therapeutic target for vitiligo and showed that treatment with PD-L1 fusion protein reversed depigmentation in a Pmel-1 vitiligo mouse model (56). Around 60% of the original pigment loss was restored upon treatment. PD-L1 fusion protein reversed depigmentation in Pmel-1 vitiligo mice *via* a marked increase in regulatory T cells in the skin and a decrease in melanocyte-reactive T cells. Fortunately, no significant side effects were observed, among which skin oncogenesis, indicating that targeting the PD-1/PD-L1 axis can be effective as a treatment strategy for T cell-induced vitiligo. The suppression of depigmentation was relatively stable until 8 weeks after last treatment, but, none of the mice regained permanent repigmentation. This limited (long-term) repigmentation in Pmel-1 vitiligo mice might be due to loss of a pigment cell reservoir, suggesting that the presence of a viable melanocyte reservoir might be crucial for the efficacy of PD-L1 fusion protein and other immune suppressive therapies. More specifically, human vitiligo lesions in hairless skin and those containing white hair do not have a strong capacity for repigmentation (57). PD-1/PD-L1 therapy, therefore, might be most effective in an early or progressive stage of vitiligo, supported by the observation that disease duration negatively affects prognosis to treatment (58). Nevertheless, patients with long-duration vitiligo may repigment well, as long as their lesions contain pigmented hair. Patients with active vitiligo might therefore be the best responders to immunomodulating agents, whereas patients with long-duration disease might need additional melanocyte transplantation (57).

Concomitantly, UV phototherapy, especially narrow-band ultraviolet B (NB-UVB) therapy, might be necessary to obtain more complete repigmentation. Among current topical and systemic treatment modalities, NB-UVB phototherapy has emerged as one of the safest and most effective therapies in vitiligo (59). Therapeutically enhancing immune checkpoint signaling may induce peripheral tolerance, yet melanocytes might still need additional stimulation to restore skin pigmentation. At the same time, NB-UVB therapy likely affects PD-L1 expression. UV-B radiation was shown to induce PD-L1 expression in human melanoma cell lines in an NF-κB-dependent manner (60). Similarly, UV-B treatment induced NF-κB activation in human primary epidermal melanocytes, implying PD-L1 upregulation is a conserved stress response to UV exposure in human melanocytes and melanoma cells that can inhibit effector T cell activity. Altogether, combined use of local agonistic PD-1/PD-L1 treatment and NB-UVB therapy seems promising in inducing local melanocyte tolerance and significant repigmentation.

Better understanding of the PD-1/PD-L1 may provide clues on how this pathway can most effectively be targeted in vitiligo. Topical treatment modalities, optionally with laser assisted delivery, or local injection are preferred over systemic therapy, partly to avoid systemic tolerance. Vitiligo patients show local autoreactivity resulting in depigmented skin patches, while tolerance to melanocyte antigens seems to predominate in other parts of the skin, leaving those pigmented. The feasibility of local vitiligo treatment, however, depends on the vitiligo disease extent. At the same time, as mentioned before, therapy is preferably given at an early disease stage, to acquire full melanocyte tolerance, long-term efficacy and possibly curation.

Local immunotherapy might decrease immunosurveillance, carrying a potential risk for oncogenesis. Vitiligo patients, however, have a 3-fold less risk of developing melanoma during life (19, 20). We, therefore, hypothesize that agonistic PD-1/PD-L1 therapy will increase the probability of melanoma development to the risk of healthy, control individuals. Nonetheless, clinicians should be aware of potential tolerance to, e.g., transformed melanocytes or keratinocytes

## Discussion

Mounting evidence has shown that signaling *via* the PD-1/PD-L1 axis is commonly involved in melanoma immune evasion and targeting these immune checkpoint molecules can reactivate potent effector immune responses against the tumor. The growing appreciation of similarities between vitiligo (autoimmunity) and melanoma (tumor immunity), has led to increased interest in PD-1/PD-L1 as promising targets for future immunotherapies in human vitiligo.

**Figure 1:**
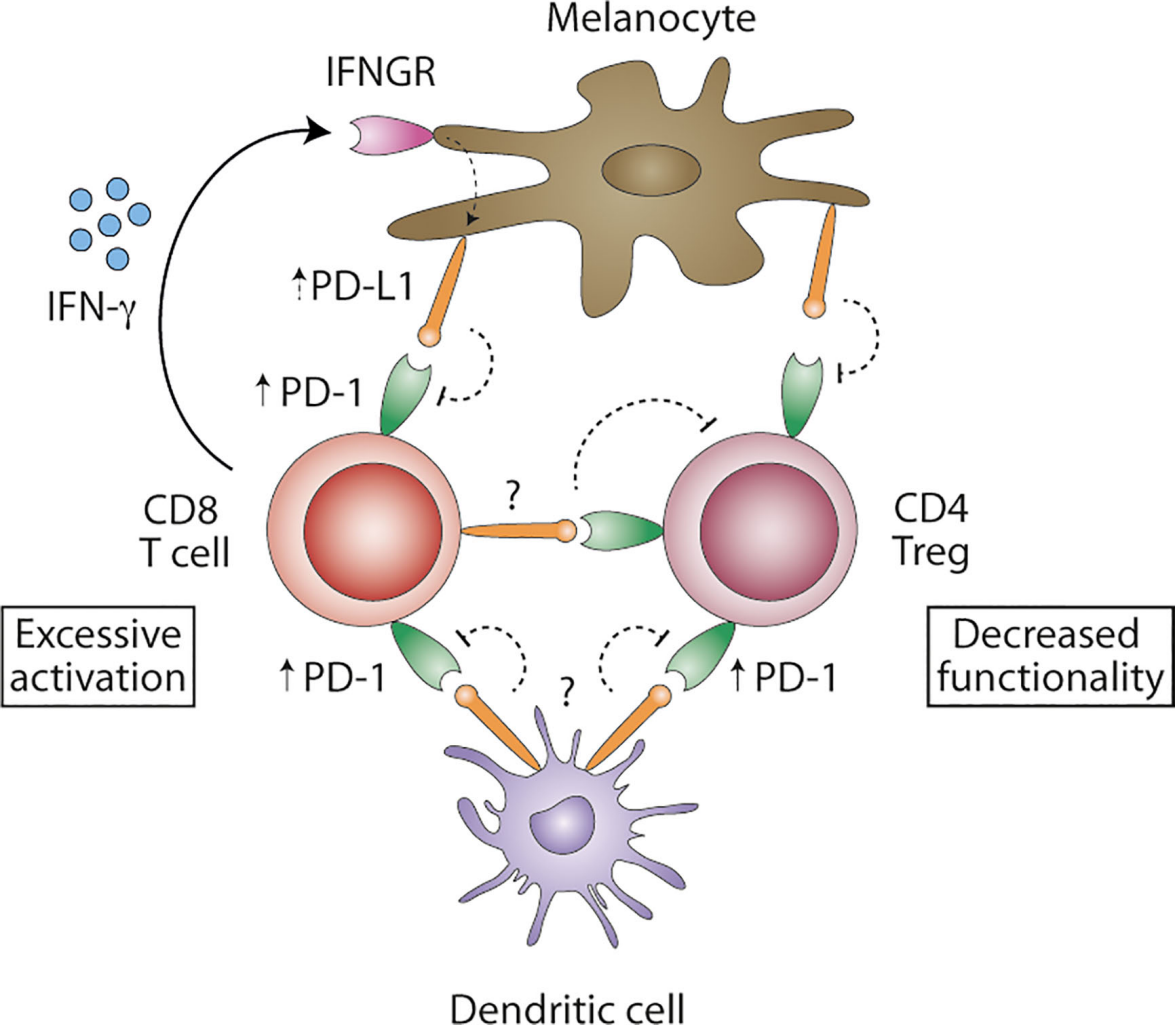
Proposed model of PD-1/PD-L1 signaling in vitiligo. Blood regulatory CD4^+^ and cytotoxic CD8^+^ T cells show increased levels of PD-1 protein. IFN-γ-producing CD8^+^ T cell are abundantly present in affected skin and may induce PD-L1 expression on melanocytes, albeit possibly ineffective in inhibiting autoreactive T cells. Consequently, melanocyte-reactive CD8^+^ T cells remain activated. PD-L1 expression on DCs remains unstudied in vitiligo and might be important in effectively inhibiting autoreactive CD8^+^ T cells. PD-L1^+^ DC might inhibit not only T cell priming in the tumor-draining lymph nodes, but also T cell effector function. PD-L1 expression *in situ* remains unclear but might be expressed by melanocytes, DC or melanocyte-specific T cells, turning PD-1^+^ regulatory CD4^+^ T cells to functional exhaustion. Overcoming excessive activation of autoreactive CD8^+^ T cells and decreased functionality of regulatory CD4^+^ T cells in vitiligo might be achieved by interfering with PD-1/PD-L1 signaling.

Although not fully confirmed so far, PD-1/PD-L1 seems to be involved in loss of peripheral tolerance in human vitiligo, with PD-1 being expressed on regulatory and CD8^+^ T cells, and *in vivo* PD-L1 protein therapy reversing depigmentation in murine vitiligo. At the same time, however, PD-1/PD-L1 expression on other cell types, among which dendritic cells (DCs), B cells, keratinocytes and fibroblasts, remain understudied and might be relevant. Recently, efficacy of anti-PD-1/PD-L1 therapy in cancer was attributed to PD-L1 expression on DCs, rather than tumor cells (61–64). PD-1/PD-L1 blockade enhanced maturation of DC in tumor-draining lymph nodes and increased T cell priming (61, 62). In **Figure 1**, we propose a model of how PD-1/PD-L1 signaling in vitiligo maintains an inflammatory environment. Vitiligo patients show increased PD-1 expression on peripheral regulatory CD4^+^ and cytotoxic CD8^+^ T cells. Concomitantly, PD-1^+^ mononuclear cells have been identified in (peri-)lesional vitiligo skin.

Though (peripheral) T cells have been demonstrated to be PD-1^+^, PD-L1 expression remains largely understudied. PD-L1 can be expressed by melanocytes, especially in the case of inflammatory responses (43). IFN-γ-producing CD8^+^ T cell are abundantly present in affected skin, but to date, (cytokine-induced) PD-L1 expression by melanocytes in (peri)lesional vitiligo skin remains unclear. If expressed, PD-L1^+^ melanocytes seem to be ineffective in inhibiting melanocyte-specific T cells. Concomitantly, PD-L1 expression on myeloid cells, among which DC, in vitiligo skin has received no attention thus far. PD-L1^+^ myeloid cells might be important in inhibiting autoreactive T cells as well. Future studies should clarify PD-L1 expression levels *in situ* to determine if these are diminished in human vitiligo and, thus, insufficiently inhibit autoreactive CD8^+^ T cells. Finally, it has been hypothesized that PD-L1-expressing melanocyte-specific T cells cause exhaustion of PD-1^+^ regulatory T cells, hence leading to uncontrolled autoimmunity (45). It is, therefore, important to study if PD-L1^+^ cells decrease peripheral tolerance by inhibiting CD4^+^ regulatory T cell function. Most importantly, however, are the PD-1 expression levels on skin-resident T cells, for skin autoimmunity appears locally and PD-L1-induced peripheral tolerance is only relevant when PD-1^+^ T cells are present.

Given that PD-1 is expressed by both peripheral regulatory CD4^+^ and cytotoxic CD8^+^ T cells in vitiligo patients, we reasoned that agonistic PD-1/PD-L1 therapy might suppress melanocyte-reactive T cells, but concomitantly suppress regulatory T cells. Counterintuitively, injection of PD-L1 fusion protein in Pmel-1 vitiligo mice led to a marked enrichment of regulatory T cells in the skin and a reduction in effector T cells (56), implying PD-L1 negatively affects autoreactive T cells, but not regulatory T cells. PD-1 expression by regulatory T cells, however, was left unstudied in this mouse model. This suggests that effector T cells were the main PD-1^+^ T cell population. Considering the presence of PD-1^+^ CD4^+^ regulatory T cells in blood of vitiligo patients, PD-L1 fusion protein might give different results in the human setting, justifying further research on the effect of increased PD-1/PD-L1 signaling on both T cell subsets to determine treatment outcome. Since topical or local treatment modalities are preferred, we hypothesize that PD-1 expression by skin-resident regulatory or memory T cells are most relevant for therapy outcome, rather than peripheral PD-1 expression.

With more knowledge becoming available on PD-1 and PD-L1 expression levels among different immune cell subsets, targeting this pathway might induce effective long-term melanocyte-specific tolerance in human vitiligo.

## Author Contributions

MW wrote the manuscript with input from all authors. All authors contributed to the article and approved the submitted version.

## Conflict of Interest

CM was employed by ISA Pharmaceuticals.

The authors declare that the research was conducted in the absence of any commercial or financial relationships that could be construed as a potential conflict of interest.
